# William Gould, M. D. (Obituary)

**Published:** 1882-04

**Authors:** 


### William Gould, M. D.

The subject of this notice graduated at the Buffalo Medical College in 1850. He commenced the study of medicine long after reaching mature years, and on receiving his degree settled in this city, where his entire professional life has been passed. Dr. Gould was a very successful practitioner, possessing a quick insight into disease, and clear and practical methods of treatment. Endowed with more than ordinary natural ability, he directed his energies earnestly to his professicn and achieved merited success. As a parliamentarian Dr. Gould was by far the most ready and apt of any who have been honored with the position of President of the Erie County Medical Society and the Buffalo Medical Association. In these positions, and as President of the Buffalo Free Dispensing Association, he exhibited executive ability rarely met with in the profession. At a meeting of the Erie County Medical Society, held March 21, 1882, eulogistic remarks were made by several of the members, and the following memorial unanimously adopted:

### MEMORIAL.

In the early dawn of Monday, March 20, 1882, passed from earth to the rest of Paradise, William Gould, M. D., aged 70 years and nine months.

The profession of Buffalo and Erie county, assembled in special session, desire to express their deep and sincere sorrow in view of the sad event which takes away another of our oldest and most respected members.

We gratefully recognize that this society is thus deprived of a strong and earnest supporter upon whom the highest honors in its gift have been bestowed, and from whom it has received in return all that zeal and energy could accomplish. We appreciate the ability and industry which he brought to his life work, and the delicate sense of honor which characterized him in all his professional and social relations. Endowed with more than ordinary natural ability, he guided his professional labors with the most mature judgment, and under difficulties and amid dangers exhibited a self-reliance which ensured success. As a member of the community he was widely known and universally respected, and among his patrons greatly beloved. The divine principle of charity for the poor and the afflicted was exhibited in his daily life and gained as a reward the consciousness of faithful stewardship in his profession.

Passing away to his eternal rest in the fulness of years and with the respect and love of the profession to which the best of his days were given, Dr. Gould leaves a record of fidelity to duty which this Society gratefully records. It is therefore

*Resolved*, That the members of this Society attend the funeral in a body; that a copy of this memorial be transmitted to the family, and also engrossed in the minutes.

